# Clinical features and prognostic factors of intensive and non-intensive 1014 COVID-19 patients: an experience cohort from Alahsa, Saudi Arabia

**DOI:** 10.1186/s40001-021-00517-7

**Published:** 2021-05-24

**Authors:** Saad Alhumaid, Abbas Al Mutair, Zainab Al Alawi, Khulud Al Salman, Nourah Al Dossary, Ahmed Omar, Mossa Alismail, Ali M. Al Ghazal, Mahdi Bu Jubarah, Hanan Al Shaikh, Maher M. Al Mahdi, Sarah Y. Alsabati, Dayas K. Philip, Mohammed Y. Alyousef, Abdulsatar H. Al Brahim, Maitham S. Al Athan, Salamah A. Alomran, Hatim S. Ahmed, Haifa Al-Shammari, Alyaa Elhazmi, Ali A. Rabaan, Jaffar A. Al-Tawfiq, Awad Al-Omari

**Affiliations:** 1 Administration of Pharmaceutical Care, Alahsa Health Cluster, Ministry of Health, Rashdiah Street, P. O. Box 12944, Alahsa, 31982 Saudi Arabia; 2 Research Center, Almoosa Specialist Hospital, Alahsa, Saudi Arabia; 3 School of Nursing, Wollongong University, Wollongong, Australia; 4 College of Nursing, Princess Norah Bint Abdul Rahman University, Riyadh, Saudi Arabia; 5 Division of Allergy and Immunology, College of Medicine, King Faisal University, Alahsa, Saudi Arabia; 6 Nursing Department, Al Jaber Hospital for Eye, Ear, Nose and Throat, Ministry of Health, Al-Hofuf, Saudi Arabia; 7 General Surgery Department, Alomran General Hospital, Alahsa, Saudi Arabia; 8 Internal Medicine Department, Alomran General Hospital, Alahsa, Saudi Arabia; 9 Pharmacy Department, King Faisal General Hospital, Alahsa, Saudi Arabia; 10 Infection Prevention and Control Department, Prince Saud Bin Jalawi Hospital, Alahsa, Saudi Arabia; 11 Nursing Department, Maternity and Children Hospital, Alahsa, Saudi Arabia; 12 Nursing Education Department, Maternity and Children Hospital, Alahsa, Saudi Arabia; 13 Administration of Academic Affairs and Research, Ministry of Health, Alahsa, Saudi Arabia; 14 Pharmacy Department, King Fahad Hofuf Hospital, Alahsa, Saudi Arabia; 15 Planning and Research Department, Ministry of Health, Alahsa, Saudi Arabia; 16 Histopathology Department, King Saud Medical City, Riyadh, Saudi Arabia; 17 Intensive Care Unit Department, Dr. Sulaiman Al Habib Medical Group, Riyadh, Saudi Arabia; 18 Molecular Diagnostics Laboratory, Johns Hopkins Aramco Healthcare, Dhahran, Saudi Arabia; 19 Infectious Disease Unit, Specialty Internal Medicine, Johns Hopkins Aramco Healthcare, Dhahran, Saudi Arabia; 20 Department of Medicine, Indiana University School of Medicine, Indianapolis, IN USA; 21 Department of Medicine, Johns Hopkins University School of Medicine, Baltimore, MD USA; 22 College of Medicine, Alfaisal University, Riyadh, Saudi Arabia; 23 Research Center, Dr. Sulaiman Al Habib Medical Group, Riyadh, Saudi Arabia

**Keywords:** Admission, Clinical, Characteristics, COVID-19, ICU, Intensive, Care, Critical, Mortality, Outcomes, SARS-CoV-2, Saudi Arabia

## Abstract

**Background:**

COVID-19 is a worldwide pandemic and has placed significant demand for acute and critical care services on hospitals in many countries.

**Objectives:**

To determine the predictors of severe COVID-19 disease requiring admission to an ICU by comparing patients who were ICU admitted to non-ICU groups.

**Methods:**

A cohort study was conducted for the laboratory-confirmed COVID-19 patients who were admitted to six Saudi Ministry of Health’s hospitals in Alahsa, between March 1, 2020, and July 30, 2020, by reviewing patient’s medical records retrospectively.

**Results:**

This cohort included 1014 patients with an overall mean age of 47.2 ± 19.3 years and 582 (57%) were males. A total of 205 (20%) of the hospitalized patients were admitted to the ICU. Hypertension, diabetes and obesity were the most common comorbidities in all study patients (27.2, 19.9, and 9%, respectively). The most prevalent symptoms were cough (47.7%), shortness of breath (35.7%) and fever (34.3%). Compared with non-ICU group, ICU patients had older age (*p* ≤ 0.0005) and comprised a higher proportion of the current smokers and had higher respiratory rates (*p* ≤ 0.0005), and more percentage of body temperatures in the range of 37.3–38.0 °C (*p* ≥ 0.0005); and had more comorbidities including diabetes (*p* ≤ 0.0005), hypertension (*p* ≥ 0.0005), obesity (*p* = 0.048), and sickle cell disease (*p* = 0.039). There were significant differences between the non-ICU and ICU groups for fever, shortness of breath, cough, fatigue, vomiting, dizziness; elevated white blood cells, neutrophils, alanine aminotransferase and alkaline aminotransferase, lactate dehydrogenase, and ferritin, and decreased hemoglobin; and proportion of abnormal bilateral chest CT images (*p* < 0.05). Significant differences were also found for multiple treatments (*p* < 0.05). ICU patients group had a much higher mortality rate than those with non-ICU admission (*p* ≤ 0.0005).

**Conclusion:**

Identifying key clinical characteristics of COVID-19 that predict ICU admission and high mortality can be useful for frontline healthcare providers in making the right clinical decision under time-sensitive and resource-constricted environment.

## Background

A novel coronavirus was recognized as the cause of a cluster of pneumonia cases before the end of 2019 and spread throughout China and elsewhere [1]. World Health Organization (WHO) has officially named the virus severe acute respiratory syndrome coronavirus 2 (SARS-CoV-2) and the disease corona virus disease 2019 (COVID-19) [2]. On January 30, 2020, the WHO declared the COVID-19 outbreak a public health emergency of international concern and, in March 2020, began to characterize it as a pandemic, in order to highlight the seriousness of the situation and urge all countries to take action in detecting infection and preventing spread. The main transmission route of SARS-CoV-2 is through the air (airborne transmission) and on a daily basis the number of deaths associated with COVID-19 is rapidly increasing [3]. Many countries have started the safety measures and precautions through screening people coming from overseas including China. Saudi Arabia has started early its precautions and implemented screening for travelers. A governmental move followed by several decisions made to prevent and control the outbreak. The Saudi Ministry of Health (MoH) and the Center for Disease Prevention and Control in the Kingdom have published the Coronavirus Infection Guidelines [4] along with other guidelines that should help to detect, prevent and manage the epidemic. On March 02, 2020, Saudi Arabia reported the first confirmed case of COVID-19 infection in an adult Saudi national who returned from Iran via Bahrain [5]. As of January 5, 2021, the total reported confirmed COVID-19 cases have reached more than 363,259 including more than 6,265 deaths within Saudi Arabia [6]. Studies have shown that up to 20% of the patients infected with SARS-CoV-2 develop high disease severity and need to be hospitalized [7–9]. Intensive care unit (ICU) admission is a requirement for up to 25% among those who are hospitalized [8, 10–15]. Patients infected by COVID-19 have a very broad spectrum of illness, from patients being asymptomatic to severally ill requiring an ICU bed admission [16, 17]. Thus, it is crucial to identify COVID-19 patients at risk to develop severe illness, to decrease mortality rate among those patients and improve their clinical outcomes. This report is a cohort study from six Ministry of Health’s hospitals in Alahsa Governorate, Saudi Arabia. The study describes the clinical characteristics, laboratories features as well as clinical outcomes for ICU and non-ICU hospitalized COVID-19 patients.

## Objectives

To determine the predictors of severe COVID-19 disease requiring an ICU admission by comparing patients who were ICU admitted to non-ICU groups.

## Methods

### Design

A cohort study was conducted for the laboratory-confirmed COVID-19 patients who were admitted to six Saudi Ministry of Health’s hospitals in Alahsa, namely, the King Fahad Hofuf Hospital, Prince Saud Bin Jalawi Hospital, Maternity and Children Hospital, King Faisal General Hospital, Alomran General Hospital and Aljaber Eye and ENT Hospital, between March 1, 2020, and July 30, 2020, by reviewing patient’s medical records retrospectively.

### Definitions and ICU eligibility

SARS-Cov-2 infection, defined as presenting with a fever or any respiratory symptoms, including dry cough, and especially in those with a history of travel or exposure to infected people within 2 weeks before the onset of illness since January 2020. Case definitions of confirmed human infection with SARS-Cov-2 were in accordance with the interim guidance from the WHO [18]. Patients were considered to have the symptom of fever when they had a body temperature ≥ 37.3 °C (≥ 37.3 °C orally or ≥ 37.7 °C rectally) or an elevation above a person’s known normal daily value [19].

Guidelines of the Saudi MoH on ICU Triage, Admission, and Discharge Criteria during the COVID 19 pandemic V2 [20] were used to help prepare and plan provision of ICU healthcare for patients included in this study during the ongoing pandemic.

Criteria for ICU admission of COVID-19 patients were [20]:

- Patient requiring invasive mechanical ventilation
- Patient requiring more than 2 h on non-invasive ventilation (NIV) or high-flow nasal cannula (HFNC)
- Respiratory distress
  (i) Need O_2_ > 6 L per minute to maintain SpO_2_ > 92 or PaO_2_ > 65
  (ii) Rapid escalation of oxygen requirement
  (iii) Significant work of breathing, i.e., tachypnea
- Patient with hemodynamic instability despite initial conservative fluid resuscitation
- Patient requiring vasopressor support
- Patient with a decreased level of consciousness
- Acidosis: ABG with pH < 7.3 or PCO_2_ > 50 or above patient’s baseline. Lactate > 2
- Patient with more than one organ failure
- Patient requires continuous renal preplacement therapy (CRRT) and cannot tolerate hemodialysis
- Patient with unstable vital signs not yet on vasopressors
- Patent with new ECG findings, including ischemia, arrhythmias, heart block

Only patients with a laboratory-confirmed infection were enrolled in this study. No exclusion criteria were applied for all confirmed SARS-CoV-2 cases in this study.

### Healthcare system of the Alahsa city and setting

Alahsa Governorate in the Alahsa oasis region in Eastern Saudi Arabia is the largest governorate in the Eastern Province. This region governs both urban and rural populations totaling 1.3 million people. Ministry of Health is the main public healthcare sector that provides preventive, curative, and rehabilitative healthcare services for the entire population of the Alahsa region. When a report of a suspected case of COVID-19 is generated at a primary healthcare center or a medical facility, the patient is referred to a general, specialized, secondary or tertiary care hospital and the relevant health directorates of Ministry of Health are notified. King Fahad Hofuf Hospital is a 500-bed general hospital in Hofuf. It is the biggest hospital in the city area in Alahsa. Other large referral general, specialized, secondary and/or tertiary care hospitals in Alahsa health included in this study were: Maternity and Children Hospital has a 450-bed capacity; Prince Saud Bin Jalawi Hospital, a 250-bed capacity facility; both King Faisal General Hospital and Alomran General Hospital are 200-bed capacity facilities; while Prince Sultan Cardiac Center has a capacity of 100 beds. These hospitals provide a vast number of services in several specialties and subspecialties (adult, pediatric and neonatal, cardiology, oncology, internal medicine, infectious diseases, dermatology, gastroenterology, rheumatology, hematology, radiology, geriatrics, obstetrics and gynecology, neuroscience, nephrology, orthopedics, urology, surgery, ear, nose and throat care, dental, burn and intensive care). The hospital records of patients with laboratory-confirmed SARS-Cov-2 infection treated at these hospitals between March 1, 2020, and July 30, 2020, were reviewed retrospectively.

### Main outcome measures

A Microsoft Excel data sheet listing the demographic, clinical, radiography and laboratory variables, and treatment outcomes, were used for the data collection. Variables included patients’ information (i.e., case identifying number, sex, age, nationality, level of education, occupation, use of tobacco and exposure history), information on the name of the hospital, data on time from exposure to the onset of illness, and time from illness onset to first hospital admission, patient’s medical history and/or co-morbid conditions, signs and symptoms of SARS-CoV-2 illness, time of symptom onset, clinical symptoms, laboratory abnormalities, chest CT findings, medications administered to the patient and treatment outcomes (i.e., hospitalization, discharged transferred or died). Information sources were medical files, electronic health information records and laboratories reports of COVID-19 patients. Patients were stratified based on ICU admission status.

### Data management and analysis

Descriptive statistics were used to describe the data. For categorical variables, frequencies and percentages were reported. Differences between groups were analyzed using the Chi-square (*χ*^*2*^) tests (or Fisher's exact tests for expected cell count < 5 in more than 20% of the cells). For continuous variables, mean and standard deviation were used to summarize the data and analyses were performed using Student's *t*-tests (Mann–Whitney U test if data are not normally distributed). An a priori two-tailed level of significance was set at 0.05. Statistical analyses were performed using Microsoft Excel 2010 (Microsoft Corp., Redmond, USA) and IBM SPSS Statistics software, version 22.0 (IBM Corp., Armonk, NY, USA).

### Ethics considerations

This study obtained approval from the Institutional Review Board in King Fahad Hofuf Hospital in Alahsa Health Cluster [IRB KFHH No. (H-05-HS-065)], and was performed according to the Helsinki Declaration. Throughout the study unique patient codes to each study participant were issued to maintain anonymity and confidentiality.

## Results

### Demographic and clinical characteristics

**Fig. 1:**
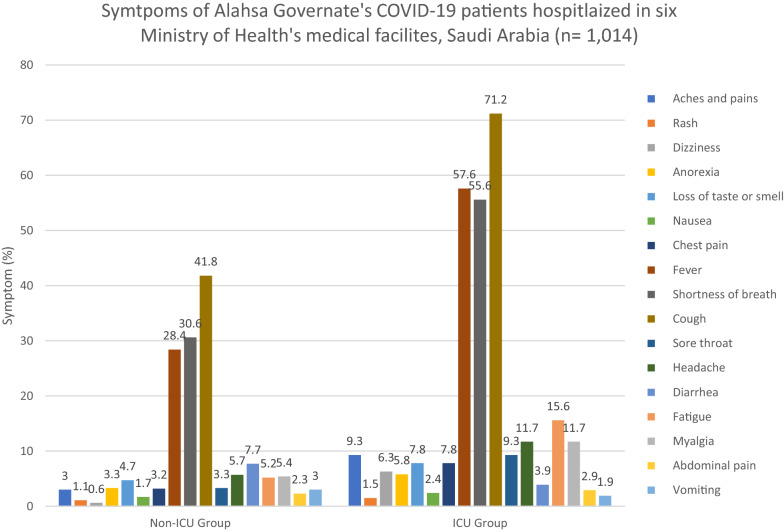
Symptoms of SARS-CoV-2 hospitalized patients in six Ministry of Health’s hospitals in Alahsa, Saudi Arabia (*n* = 1014)

The study population included 1,014 hospitalized patients with confirmed COVID-19. The overall mean age of the hospitalized SARS-CoV-2 cohort was 47.2 ± 19.3 years, ranging from 1 month to ≥ 90 years. A total of 57.4% (*n* = 582) of the patients were males and 88.8% (n = 900) were Saudi citizens. About 52.2% (*n* = 529) of the patients received an educational level of primary to secondary and 2.1% (*n* = 21) were healthcare providers. Almost 66 percent (*n* = 668) had contact with positive confirmed SARS-CoV-2 cases and 17.8% (*n* = 180) had familiar or cluster infections. Only one patient had a history of travel to a country with a high risk of COVID-19 transmission. The remaining percentage of the patients (14.1%; *n* = 144) had unknown mode of infection. Hypertension, diabetes and obesity (BMI ≥ 30 kg/m^2^) were the most common comorbidities in all study patients (27.2, 19.9, and 9%, respectively) (Table 1). As shown in Fig. 1, the most prevalent symptoms at onset of illness were cough (47.7%; *n* = 484), shortness of breath (35.7%; *n* = 362) and fever (34.3%; *n* = 348). Minor symptoms were headache (6.9%; *n* = 70); loss of taste or smell (5.3%; *n* = 54); gastrointestinal symptoms [diarrhea (6.9%; *n* = 70), vomiting (2.8%; *n* = 28), abdominal pain (2.5%; *n* = 25), and nausea (1.9%; *n* = 19)]; anorexia (3.8%; *n* = 39); and rash (1.1%; *n* = 12).

**Table 1:** Demographic and clinical characteristics of 1,014 patients with COVID-19 hospitalized in Alahsa Governorate, Saudi Arabia, stratified by ICU admission

Characteristic	All (*n* = 1014)	Non-ICU (*n* = 809)	ICU (*n* = 205)	*p*-value
Demographics				
Age, mean ± SD, years	47.2 ± 19.3 (1–104)	45.3 ± 19.3 (1–104)	52.9 ± 17.3 (5–95)	≤ 0.0005
Distribution				
0–10 years	28 (2.8%)	20 (2.5%)	8 (3.9%)	≤ 0.0005
11–20 years	37 (3.6%)	33 (4.2%)	4 (1.9%)	
21–30 years	126 (12.5%)	110 (13.6%)	16 (7.8%)	
31–40 years	214 (21.1%)	195 (24.1%)	19 (9.3%)	
41–50 years	164 (16.2%)	137 (16.9%)	27 (13.2%)	
51–60 years	186 (18.3%)	155 (19.2%)	31 (15.1%)	
61–70 years	144 (14.2%)	108 (13.3%)	36 (17.6%)	
71–80 years	65 (6.4%)	32 (3.9%)	33 (16.1%)	
81–90 years	37 (3.6%)	10 (1.2%)	27 (13.2%)	
≥ 90 years	13 (1.3%)	9 (1.1%)	4 (1.9%)	
Gender				
Male	582 (57.4%)	466 (57.6%)	116 (56.5%)	0.858
Female gender	432 (42.6%)	343 (42.4%)	89 (43.5%)	
Nationality				
Saudi	900 (88.8%)	721 (89.1%)	179 (87.3%)	0.886
Non-Saudi	114 (11.2%)	88 (10.9%)	26 (12.7%)	
Occupation				
Healthcare worker	21 (2.1%)	16 (2%)	5 (2.4%)	0.755
Non-healthcare worker	993 (97.9%)	793 (98%)	200 (97.6%)	
Smoking status				
Not a smoker	920 (90.7%)	786 (97.2%)	134 (65.4%)	0.223
Past smoker	35 (3.4%)	6 (0.7%)	29 (14.1%)	
Current smoker	59 (5.9%)	17 (2.1%)	42 (20.5%)	
Hospital				
King Fahad Hofuf Hospital	300 (29.6%)	249 (30.8%)	51 (24.9%)	≤ 0.0005
Aljabr Eye and ENT Hospital	218 (21.5%)	174 (21.5%)	44 (21.5%)	
Alomran General Hospital	200 (19.7%)	150 (18.5%)	50 (24.4%)	
King Faisal General Hospital	163 (16.1%)	152 (18.8%)	11 (5.4%)	
Maternity and Children Hospital	71 (7%)	55 (6.8%)	16 (7.8%)	
Prince Saud Bin Jalawi Hospital	62 (6.1%)	29 (3.6%)	33 (16%)	
Educational level				
Illiterate	146 (14.4%)	61 (7.5%)	85 (41.5%)	0.06
Primary to secondary	529 (52.2%)	449 (55.5%)	80 (39%)	
University	172 (17%)	153 (18.9%)	19 (9.3%)	
Unknown	167 (16.4%)	146 (18.1%)	21 (10.2%)	
Exposure history				
Contact with positive confirmed case	668 (65.9%)	548 (67.7%)	120 (58.5%)	0.062
Familiar/cluster infections	180 (17.8%)	149 (18.4%)	31 (15.1%)	
Hospital staff	21 (2.1%)	17 (2.1%)	4 (1.9%)	
From outside Saudi	1 (0.1%)	1 (0.1%)	0	
Unknown	144 (14.1%)	94 (11.6%)	50 (24.4%)	
Highest temperature (°C):				
< 37.3	467 (46.1%)	414 (51.3%)	53 (25.8%)	≤ 0.0005
37.3–38.0	338 (33.3%)	226 (27.9%)	112 (54.7%)	
38.01–39.0	147 (14.5%)	116 (14.3%)	31 (15.1%)	
> 39.0	62 (6.1%)	53 (6.5%)	9 (4.4%)	
Respiratory rate > 24 breaths per min	312 (30.8%)	168 (20.8%)	144 (70.2%)	≤ 0.0005
Comorbidities				
Diabetes	202 (19.9%)	142 (17.5%)	60 (29.3%)	≤ 0.0005
Hypertension	276 (27.2%)	165 (20.4%)	111 (54.1%)	≤ 0.0005
Obesity (BMI ≥ 30 kg/m^2^)	91 (9%)	65 (8%)	26 (12.7%)	0.048
Asthma	26 (2.6%)	15 (1.8%)	11 (5.4%)	0.179
Coronary artery disease	16 (1.6%)	7 (0.9%)	9 (4.4%)	0.778
Thalassemia	3 (0.3%)	0	3 (1.5%)	0.222
Chronic kidney disease	5 (0.5%)	0	5 (2.4%)	0.592
Liver disease	1 (0.1%)	0	1 (0.5%)	0.778
Cerebrovascular disease	6 (0.6%)	3 (0.4%)	3 (1.5%)	0.127
Cancer	5 (0.5%)	1 (0.1%)	4 (1.9%)	0.691
Cardiomyopathies	3 (0.3%)	1 (0.1%)	2 (1%)	0.395
Heart failure	2 (0.2%)	0	2 (1%)	0.605
Ischemic heart disease	12 (1.2%)	3 (0.4%)	9 (4.4%)	0.520
Sickle cell disease	31 (3%)	17 (2.1)	14 (6.8%)	0.039
G6PD deficiency	14 (1.4%)	11 (1.3%)	3 (1.5%)	0.621
Pregnant	18 (1.8%)	10 (1.2%)	8 (3.9%)	0.379
Asymptomatic	152 (15%)	148 (18.3%)	4 (1.9%)	≤ 0.0005
Symptoms				
Fever	348 (34.3%)	230 (28.4%)	118 (57.6%)	≤ 0.0005
Shortness of breath	362 (35.7%)	248 (30.6%)	114 (55.6%)	≤ 0.0005
Cough	484 (47.7%)	338 (41.8%)	146 (71.2%)	≤ 0.0005
Aches and pains	43 (4.2%)	24 (3%)	19 (9.3%)	0.139
Loss of taste or smell	54 (5.3%)	38 (4.7%)	16 (7.8%)	0.562
Nausea	19 (1.9%)	14 (1.7%)	5 (2.4%)	0.170
Chest pain	42 (4.1%)	26 (3.2%)	16 (7.8%)	0.258
Sore throat	46 (4.5%)	27 (3.3%)	19 (9.3%)	0.053
Headache	70 (6.9%)	46 (5.7%)	24 (11.7%)	0.063
Diarrhea	70 (6.9%)	62 (7.7%)	8 (3.9%)	0.077
Fatigue	74 (7.3%)	42 (5.2%)	32 (15.6%)	0.011
Myalgia	68 (6.7%)	44 (5.4%)	24 (11.7%)	0.153
Abdominal pain	25 (2.5%)	19 (2.3%)	6 (2.9%)	0.102
Vomiting	28 (2.8%)	24 (3%)	4 (1.9%)	0.013
Anorexia	39 (3.8%)	27 (3.3%)	12 (5.8%)	0.399
Rash	12 (1.1%)	9 (1.1%)	3 (1.5%)	0.470
Dizziness	18 (1.8%)	5 (0.6%)	13 (6.3%)	0.024
Time from exposure to the onset of illness (days)	5.39 ± 3.8 (1–15)	5.98 ± 3.96 (1–14)	4.49 ± 3.5 (2–15)	0.041
Time from illness onset to first hospital admission (days)	5.99 ± 3.9 (1–30)	5.97 ± 3.7 (1–22)	6.04 ± 4.45 (1–30)	0.646

Data are presented as number (%), or mean ± SD and (minimum–maximum)

*COVID-19* coronavirus disease 2019, *ICU* intensive care unit, *G6PD* Glucose-6-phosphate dehydrogenase, *BMI* body mass index, *SD* standard deviation

A total of 205 (20.2%) of the hospitalized patients were admitted to ICU. As illustrated in Table 1, those patients admitted to the ICU were more likely to have been older in age (45.3 vs 52.9 years; *p* ≤ 0.0005); tenfold more likely to be current smokers compared to the non-ICU group (2.1 vs 20.5%); and suffered the highest body temperatures more in the range of 37.3–38.0 °C; *p* ≤ 0.0005. Respiratory rate of > 24 breaths per minute (bpm) was a lot more common in the ICU patients (20 vs 70%; *p* ≤ 0.0005). Comorbidities of hypertension, diabetes and obesity were statistically significant between the two groups (hypertension: 20.4% for non-ICU group vs 54.1% for ICU group, *p* ≤ 0.0005; diabetes: 17.5% for non-ICU group vs 29.3% for ICU group, *p* ≤ 0.0005; and obesity: 8% for non-ICU group vs 12.7% for ICU group, *p* = 0.048). Fourteen (6.8%) of the 205 cases in the ICU group had a medical comorbidity of sickle cell disease (*p* = 0.039). Compared with the patients in the ICU group, a greater percentage of patients in the non-ICU group presented to the hospital with the COVID-19 infection asymptomatically (18.3 vs 1.9%, *p* ≤ 0.0005). Also, ICU patients presented with more cough (71 vs 41%, *p* ≤ 0.0005), fever (28 vs 57%, *p* ≤ 0.0005), shortness of breath (30 vs 55%, *p* = 0.000) fatigue (5 vs 15%, *p* = 0.011), and dizziness (0.6 vs 6%, *p* = 0.024). ICU patients experienced a little less vomiting compared to the non-ICU group (3 vs 1.9%, *p* = 0.011) and shorter time from exposure to the onset of illness (5.9 vs 4.5 mean days; *p* = 0.041).

### Laboratory and chest CT findings

Patients admitted to the ICU in this study were more likely to present with higher levels of the following: white blood cell count (6.9 × 10^9^/L vs 10.3 × 10^9^/L; *p* ≤ 0.0005), neutrophil count (3.2 × 10^9^/L vs 4.9 × 10^9^/L; *p* ≤ 0.0005), alanine aminotransferase and aspartate aminotransferase (43 vs 92 U/L, *p* ≤ 0.0005; and 66 vs 109 U/L, *p* ≤ 0.0005; respectively), lactate dehydrogenase level (14 U/L vs 22 U/L; p ≤ 0.0005), and ferritin (502 vs 1,647 µg/L; *p* ≤ 0.0005). ICU group had lower hemoglobin levels (12.5 vs 11.3 g/dL; *p* ≤ 0.0005); and shown lower rate of normal chest CT images (35 vs 5, *p* ≤ 0.0005), and more major bilateral lung abnormalities (35 vs 69%; *p* ≤ 0.0005). Other investigations of the cohort are indicated in Table 2.

**Table 2:** Laboratory and chest radiography findings in 1,014 patients with COVID-19 admitted to six hospitals in Alahsa Governorate, Saudi Arabia, stratified by ICU admission

Characteristic	All (*n* = 1014)	Non-ICU (n = 809)	ICU (*n* = 205)	*p*-value
Laboratory findings				
White blood cell count (× 10^9^/L)^a^	7.7 ± 4.5 (1.1–42.9)	6.9 ± 3.3 (1.1–26.6)	10.3 ± 6.7 (1.4–42.9)	≤ 0.0005
White blood cell count (× 10^9^/L) (No (%))^a^				
< 4	150 (14.8%)	127 (15.7%)	23 (11.3%)	≤ 0.0005
4–10	651 (64.2%)	555 (68.6%)	96 (46.8%)	
> 10	213 (21%)	127 (15.7%)	86 (41.9%)	
Neutrophil count (× 10^9^/L)^b^	3.4 ± 3.2 (0.01–9.7)	3.2 ± 3.3 (0.01–9.6)	4.9 ± 3.6 (0.1–9.7)	≤ 0.0005
Lymphocyte count (× 10^9^/L)^b^	1.3 ± 1.6 (0.3–8.4)	1.2 ± 1.6 (0.34–8.4)	1.3 ± 1.5 (0.3–6.2)	0.278
Lymphocyte count (× 10^9^/L) (No (%))^a^				
< 1.0	195 (19.2%)	131 (16.2%)	64 (31.2%)	0.138
≥ 1.0	819 (80.8%)	678 (83.8%)	141 (68.8%)	
Hemoglobin (g/dL)^b^	12.2 ± 2.3 (3.7–17.4)	12.5 ± 2.2 (4.5–17.4)	11.3 ± 2.3 (3.7–16.4)	≤ 0.0005
Platelet count (× 10^9^/L)^b^	285.3 ± 130.6 (8–861)	285.6 ± 123.1 (8–802)	290.2 ± 153.3 (24–861)	0.850
Platelet count (× 10^9^/L) (No (%))^a^				
< 100	80 (7.9%)	57 (7.1%)	23 (11.2%)	0.128
≥ 100	934 (92.1%)	752 (92.9%)	182 (88.8%)	
Alanine aminotransferase (U/L)^b^	55.9 ± 144.3 (3.8–2,465)	43.2 ± 84 (3.8–1,534)	92.6 ± 227.6 (5.7–2,465)	≤ 0.0005
Aspartate aminotransferase (U/L)^b^	80.1 ± 432.8 (3–10,252)	66.8 ± 463.5 (3–10,252)	109.5 ± 283 (7.3–2,870)	≤ 0.0005
Aspartate aminotransferase (U/L) (No (%))^a^				
< 40	704 (69.4%)	592 (73.2%)	112 (54.6%)	≤ 0.0005
≥ 40	310 (30.6%)	217 (26.8%)	93 (45.4%)	
Potassium (mmol/L)^b^	4.4 ± 0.7 (2.8–8.1)	4.4 ± 0.7 (2.8–8.1)	4.5 ± 0.7 (2.9–7.5)	0.132
Sodium (mmol/L)^b^	137.9 ± 5.7 (57.8–163)	137.7 ± 5.4 (57.8–163)	138.8 ± 6.5 (127–163)	0.960
Creatinine (μmol/L)^b^	89 ± 96.5 (5.2–927)	84.5 ± 92.7 (5.7–927)	92.5 ± 81.4 (5.2–645)	0.081
Creatinine (μmol/L) (No (%)^a^				
≤ 107	833 (82.1%)	673 (83.2%)	160 (78.1%)	0.002
> 107	181 (17.9%)	136 (16.8%)	45 (21.9%)	
Creatinine kinase (U/L)^b^	257.6 ± 821 (2.2–9,716)	204 ± 673.4 (2.2–9,716)	497.4 ± 1,314 (9–9,716)	0.098
Creatinine kinase (U/L) (No (%))^a^				
≤ 198	680 (67.1%)	557 (68.8%)	123 (60.2%)	0.015
> 198	334 (32.9%)	252 (31.2%)	82 (39.8%)	
Lactate dehydrogenase (U/L)^c^	16.4 ± 20.1 (0.2–96)	14.2 ± 18.7 (0.2–96)	22.6 ± 24.2 (2.1–96)	≤ 0.0005
Lactate dehydrogenase (U/L) (No (%))^a^				
≤ 280	549 (54.1%)	479 (59.2%)	70 (34.2%)	≤ 0.0005
> 280	465 (45.9%)	330 (40.8%)	135 (65.8%)	
C-reactive protein (mg/L)^b^	16.4 ± 20.1 (0.2–96)	14.2 ± 18.7 (0.2–96)	22.6 ± 24.2 (2.1–96)	0.092
Ferritin (µg/L)^c^	845.6 ± 3,430 (2–49,813)	502.9 ± 860.5 (4.4–7,945)	1,647 ± 5,270 (2–42,969)	≤ 0.0005
ESR (mm/hour)^b^	49 ± 35.8 (1–139)	47.9 ± 35.2 (1–139)	54.3 ± 38.7 (1–130)	0.552
Chest CT images^a^				
Normal	295 (29.1%)	283 (35%)	12 (5.8%)	≤ 0.0005
Abnormal bilateral lung	427 (42.1%)	285 (35.2%)	142 (69.3%)	
Abnormal right lung	95 (9.4%)	79 (9.8%)	16 (7.8%)	
Abnormal left lung	110 (10.9%)	96 (11.9%)	14 (6.8%)	
No radiography data available	87 (8.5%)	66 (8.1%)	21 (10.2%)	

Data are presented as number (%), or mean ± SD and (minimum–maximum)

*COVID-19* coronavirus disease 2019, *ESR* erythrocyte sedimentation rate, *ICU* intensive care unit, *SD* standard deviation

^a^Chi-square (*χ*^2^) test was used to compare between non-ICU and ICU groups

^b^Mann–Whitney *U* test was used to compare between non-ICU and ICU groups

^c^Student’s *t*-test was used to compare between non-ICU and ICU groups

^*^Represents significant differences

### Pharmacotherapy agents

As shown in Table 3, the six most prescribed medications were enoxaparin (56%; *n* = 571), paracetamol (35%; *n* = 355), favipiravir (34%; *n* = 353), azithromycin (33%; *n* = 337), ceftriaxone (32%; *n* = 332), and vitamin C (31%; *n* = 322). Those patients admitted to the ICU were more likely to have been prescribed the favipiravir (54 vs 29%; *p* = 0.000), azithromycin (54 vs 27%; ≤ 0.0005), tocilizumab (29 vs 10%; *p* ≤ 0.0005), lopinavir/ritonavir (16 vs 5%; *p* ≤ 0.0005), dexamethasone (34 vs 16%; p ≤ 0.0005) and methylprednisolone (22 vs 10%; *p* ≤ 0.0005).

**Table 3:** Treatments and outcomes in 1,014 patients with COVID-19 admitted to six hospitals in Alahsa Governorate, Saudi Arabia, stratified by ICU admission

Characteristic	All (*n* = 1014)	Non-ICU (*n* = 809)	ICU (*n* = 205)	*p*-value
No pharmacotherapy was given	115 (11.3%)	88 (10.9%)	27 (13.2%)	0.338
Pharmacotherapy used				
Hydroxychloroquine	269 (26.5%)	235 (29%)	34 (16.6%)	≤ 0.0005
Azithromycin	337 (33.2%)	226 (27.9%)	111 (54%)	≤ 0.0005
Oseltamivir	88 (8.7%)	73 (9%)	15 (7.3%)	0.385
Vitamin C	322 (31.7%)	241 (29.8%)	81 (39.5%)	0.029
Vitamin D	286 (28.2%)	211 (26.1%)	75 (36.6%)	0.001
Zinc	195 (19.2%)	142 (17.5%)	53 (25.8%)	0.001
Ceftriaxone	332 (32.7%)	257 (31.8%)	75 (36.6%)	0.08
Enoxaparin	571 (56.3%)	473 (58.5%)	98 (47.8%)	0.005
Favipiravir	353 (34.8%)	241 (29.8%)	112 (54.6%)	≤ 0.0005
Thiamine	94 (9.3%)	78 (9.6%)	16 (7.8%)	0.467
Interferon beta	53 (5.2%)	33 (4.1%)	20 (9.7%)	0.002
Lopinavir/ritonavir	80 (7.9%)	47 (5.8%)	33 (16.1%)	≤ 0.0005
Dexamethasone	202 (19.9%)	131 (16.2%)	71 (34.8%)	≤ 0.0005
Hydrocortisone	84 (8.3%)	58 (7.2%)	26 (12.7%)	≤ 0.0005
Methylprednisolone	135 (13.3%)	88 (10.9%)	47 (22.9%)	≤ 0.0005
Tocilizumab	144 (14.2%)	83 (10.2%)	61 (29.7%)	≤ 0.0005
Paracetamol	355 (35%)	268 (33.1%)	87 (42.4%)	≤ 0.0005
Prognosis				
Hospitalization	87 (8.6%)	62 (7.7%)	25 (12.2%)	≤ 0.0005
Transferred	29 (2.9%)	17 (2.1%)	12 (5.8%)	
Recovered	778 (76.7%)	667 (82.4%)	111 (54.1%)	
Died	120 (11.8%)	63 (7.8%)	57 (27.8%)	

Data are presented as number (%)

*COVID-19* coronavirus disease 2019, *ICU* intensive care unit

### Treatment outcomes

Clinical outcomes of the cohort with mortality documented in 120 (11.8%) of the patients while 778 (76.7%) of the study cohort recovered. High mortality rate was associated with those that were admitted to an ICU (27.8 vs 7.8%; *p* ≤ 0.0005), if patient had comorbidities (6.3 vs 0.8%; *p* ≤ 0.0005), had cerebrovascular disease (16.7 vs 3.5%; *p* = 0.04), had diabetes (8.7 vs 1.9%; *p* ≤ 0.0005), had hypertension (7 vs 2.4%; *p* = 0.003), if patient was symptomatic (5.2 vs 0%; *p* ≤ 0.0005), if patient had respiratory rate > 24 breaths per minute (10.8 vs 0%; *p* ≤ 0.0005), older age (≥ 51 years) (25 vs 2.3%; *p* ≤ 0.0005), and those with platelet count < 100 × 10^9^/L (37 vs 10.6%; *p* ≤ 0.0005).

## Discussion

We describe the clinical characteristics, laboratory parameters, treatments and outcomes of 1,014 laboratory-confirmed COVID-19 patients admitted to six Ministry of Health’s different hospitals in Alahsa Governorate, Saudi Arabia, with a focus on ICU and non-ICU admission.

Based on previously published studies [16, 17], evidence suggests that older male patients are the most susceptible to infection of COVID-19, which was supported by our data. This might be attributed mainly to the differences in the inclusion criteria and the population groups of age in the current study or can be explained by the gender-based biological differences in the host immune response to COVID-19 infection. Additionally, gender-based biological factors underlying the immune response are important determinants of susceptibility to SARS-CoV-2 infection, disease, and mortality outcomes [21, 22]. In our study, males gender predominated admission to ICU, a finding suggested in previous reports [23–25] and in contradiction with data for other cohorts suggesting an equal proportion of ICU admission for both genders [11, 12]. However, this was probably related to different lifestyles between men and women, stronger immune response to infections in females who outlive men [26] or lower rates of healthcare service utilization by males [27]. Moreover, some authors explained this gender difference with higher rates of comorbidities among men [28], prior studies suggested a higher trend among females to follow hand hygiene [29] and preventive care [14, 30]. The mean age of ICU patients was 52 ± 17 years; similar to one small cohort in Oman State [31] and a larger cohort in Kuwait [32]; and lower than reports from large cohorts in Italy [25] and China [24]. Age appears to be the major risk factor that predicts progression of COVID-19 patients to acute respiratory distress syndrome (ARDS) and need for ICU admission [33, 34]; however, there were no statistically significant differences in age between ICU and non-ICU groups in our study. Importantly, adults of any age may develop severe disease and experience adverse outcomes, especially those with comorbidities [35].

In our cohort, most of the patients were not smokers. Nevertheless, cases who were admitted to the ICU in this study represent more than 70% of all the patients who were smoking currently. Smoking has been indicated as a risk factor in some previously published reports and predict progression and worsening from COVID-19 [24, 36].

In both non-ICU and ICU groups, a high percentage of all patients showed abnormal bilateral chest CT images consistent with pneumonia and/or ARDS. Chest CT abnormalities in COVID-19 patients as shown in other studies are often bilateral, have a peripheral distribution, and involve the lungs lower lobes [37, 38]. Though some chest CT findings may be characteristic of COVID-19, no finding can completely rule out or rule in the possibility of COVID-19. Use of chest CT for screening or diagnosis of SARS-CoV-2 is not recommended and chest radiographs should be reserved when there is an indication (e.g., catheter- or endotracheal tube-placement or a relevant clinical change is recommended [39]; this rationale is based upon the increased risk of viral shedding with procedures that require transfer out of the ICU. It is worth mentioning that among ICU patients who clinically improve, resolution of radiographic abnormalities may delay improvements in fever and hypoxia [40].

Fever, shortness of breath and cough were the most common symptoms in all patients and strong predictors for the patient’s admission to ICU. Similar findings have been reported in previous studies [16, 17]; however, fever and cough are not reliable indicators to confirm the diagnosis of COVID-19 or rule out other differential diagnoses [41], and shortness of breath usually emerges a few days after these initial symptoms. Therefore, it is difficult based on fever, cough and shortness of breath symptoms alone to distinguish COVID-19 from other bacterial or viral infections [42]. While high fever was associated with a higher likelihood of developing ARDS and admission to ICU [35], it appears to be associated with a lower mortality rate [33, 43].

In line with our results, respiratory rate has also been identified as an important predictor of ICU admission in patients with COVID-19 [9, 44, 45]. Frequent monitoring and documentation of the respiratory rate, along with education on appropriate action when the respiratory rate is fluctuating, may help to identify and manage COVID-19 patients at risk and accordingly reduce the incidence of serious adverse events.

Consistent with other studies, very common comorbidities to predict severity of COVID-19 and ICU admission were diabetes [10, 24, 25, 35, 46, 47], hypertension [10, 24, 25, 47], obesity [10, 48, 49], cardiovascular disease [24, 35, 43], chronic kidney disease [24, 25, 43], cerebrovascular disease [43, 50, 51], and sickle cell disease [52–54]. COVID-19 patients with these underlying comorbidities were more likely to develop into critically severe cases, require respiratory support treatment, and eventually be transferred to ICU.

The ICU admission rate for COVID-19 patients in our study was 20% and identical to the rates reported in a Kuwaiti study [32] and a report on the analysis of a second surge in Houston, Texas, USA [10]. Higher ICU admission rates in COVID-19 cohorts were reported; for instance, in Chinese cohorts, rates of ICU admission ranged from 7 to 26% [9, 14, 15, 43], and in a large US cohort (a total of 4,226 COVID-19 cases) reported to CDC from a total of 49 states, the District of Columbia, and three U.S. territories during February 12–March 16, 2020, ICU admission rate was 53% [55]; and there was a lower proportion of ICU admission of all hospitalized COVID-19 patients based on studies in Italy (16%) [12, 13]. Rates of ICU admission differ due to different healthcare systems, medical practice and admission criteria as well as differences in predisposing factors such as age, comorbidities and testing availability in the patients served.

We report similar results of previous studies regarding the laboratory abnormalities that have been described in severe COVID-19 patients who necessitated ICU admission including elevated white blood cells [9, 32, 56], neutrophils [9, 32, 56, 57], alanine aminotransferase and aspartate aminotransferase [9, 24, 32, 35, 45, 56, 58], creatinine [9, 32, 35, 43, 45], creatinine kinase [9, 35, 45, 56, 58], lactate dehydrogenase [9, 24, 31, 32, 35, 43, 45, 56, 58, 59], and ferritin [24, 31, 45, 56, 58, 59]. Continuous tracking of laboratory findings during the management of COVID-19 cases is crucial to early identify those patients who may progress to severe status.

We report taste and smell disturbances as a rare clinical finding in our cohort on COVID-19; however, several authors reported different findings [60–62]. A meta-analysis demonstrated a prevalence of 52.73% (95% CI: 29.64–75.23%) and 43.93% (95% CI: 20.46–68.95%) for smell and taste dysfunction among COVID-19 patients, reaching 86.60% in studies that used validated instruments [63]. In particular, these symptoms seem to be common in the early stages of the disease and in paucisymptomatic patients [64]. It was hypothesized that COVID-19 patients under‐report the frequency of taste and smell disorders, and authors suggested that those with more severe SARS-CoV-2 disease neglect such symptoms in the setting of severe respiratory disease [60, 61].

Optimal approaches to treat patients hospitalized with COVID-19 are lacking. Strategies to manage patients infected with COVID-19 are based on limited data and changes rapidly as clinical data emerge. Lack of well-defined management plan for COVID-19 disease results in the use of various treatment and adjuvant therapies during hospital stay. Many antiretroviral, anti-malarial and anti-inflammatory medications were tried on hundreds of thousands of affected patients to find effective therapeutics and to reduce the spread of this global pandemic. For mild to moderate COVID-19 cases, care is mostly supportive, with close monitoring for disease progression. Supportive measures were given to all patients in the present study which included but not limited to respiratory support, circulatory support, prevention of secondary infections, and preservation of renal, hepatic, and neurological function. In addition to the implementation of the basic principles of critical care medicine, patients in the current cohort also received pharmacologic prophylaxis for venous thromboembolism. For patients who are receiving supplemental oxygen (including those who are on high-flow oxygen and non-invasive ventilation), low-dose dexamethasone and, if available, remdesivir is/are suggested [65, 66]. However, the optimal role of remdesivir remains uncertain, and some guidelines panels recommend not using it in hospitalized patients; including the WHO; as there is no clear evidence that it improves patient-important outcomes of COVID-19 hospitalized patients such as mortality and need for mechanical ventilation. Lopinavir–ritonavir is a human immunodeficiency virus antiviral combination that has been considered for the treatment of human viral corona respiratory infections, including Middle East Respiratory Syndrome (MERS-CoV) and COVID-19 disease [67, 68]. However, use of lopinavir/ritonavir for treatment of SARS-CoV-2 in hospitalized patients is not recommended as several studies have failed to prove its efficacy [69–71]. Recently, a variety of therapeutic drug candidates with various forms of modes of action have been considered to combat extreme hyperinflammatory responses in hospitalized COVID-19 patients [72]. From a tsunami of upcoming clinical research studies, authors concluded that cytokine antagonists such as tocilizumab and sarilumab and Janus kinase-signal transducer and activator of transcription inhibitors such as ruxolitinib and baricitinib, represent potential candidates in managing severe inflammatory response or cytokine storm [72]. Convalescent plasma therapy, antibody-based therapies, mesenchymal stem cell therapies, and medicinal herbs have shown potential to combat the disease severity, lessen cytokine storm, and reduce mortality in severely ill patients with COVID-19 [72]. It is worth mentioning that initiating therapy earlier is known to be more effective [73], since systemic hyperinflammation rather than viral pathogenicity dominates later stages of SARS-CoV-2 infection. Until sufficient evidence is available, the WHO has warned against physicians and medical associations recommending or administering unproven treatments to patients with SARS-CoV-2 or people self-medicating with them.

## Limitation of the study

Cautions must be taken when generalizing of the current study findings as it has several limitations. First, the retrospective nature of the study design could have introduced some bias due to reliance on clinical medical records. Second, the follow-up was limited through July 30th, 2020, hindering the possibility of including all patients’ outcomes as some of them are still remained hospitalized. Therefore, there may have been some bias regarding the patients’ prognosis. Third, we were not able to provide details on the radiological characters of our study cohort. Finally, some of the follow-up data were missing in the medical records. Additionally, clinical follow-up data for patients after recovery from COVID-19 infection were not available which can be used to examine psychological and longer-term functional abnormalities.

## Conclusion

SARS-CoV-2 is a worldwide pandemic and has placed significant surges in demand for acute and critical care services on hospitals in many countries. Old age; body temperatures in the range of 37.3–38.0 °C; respiratory rate of > 24 bpm, diabetes; hypertension; obesity; sickle cell disease; showing symptoms of COVID-19; fever, shortness of breath, cough, fatigue, vomiting, and dizziness; elevated white blood cells, neutrophils, alanine aminotransferase and alkaline aminotransferase, lactate dehydrogenase, and ferritin; and abnormal bilateral chest CT images were associated with ICU admission. Besides, the prevalence of mortality had a strong relation with ICU admission, comorbidities; if patient had diabetes, hypertension, and cerebrovascular disease; showing symptoms of COVID-19, respiratory rate > 24 bpm, old age, and platelet count less than < 100 × 10^9^/L. Identifying key clinical characteristics of COVID-19 that predict ICU admission and high mortality rate can be useful for frontline healthcare providers in making the right clinical decision under time-sensitive and resource-constricted environment.

## Data Availability

Data are available upon request, please contact author for data requests.

## Abbreviations

COVID-19: Coronavirus disease 2019
SARS-CoV-2: Severe acute respiratory syndrome coronavirus 2
RT-PCR: Real-time reverse transcription-polymerase chain reaction
CRP: C-reactive protein
ICU: Intensive care unit
G6PD: Glucose-6-phosphate dehydrogenase
BMI: Body mass index
